# Supplementary material for: A Novel Nanobody Precisely Visualizes Phosphorylated Histone H2AX in Living Cancer Cells under Drug-Induced Replication Stress
Source: Cancers (Basel). 2021 Jul 1;13(13):3317. doi: 10.3390/cancers13133317 (PMC8267817; doi:10.3390/cancers13133317)
Supplement: Supplementary file 1 [file cancers-13-03317-s001.zip › cancers-1239565-supplementary.pdf]

Supplementary Files

A Novel Nanobody Precisely Visualizes Phosphorylated Histone H2AX in Living Cancer Cells under Drug-Induced Replication Stress

Eric Moeglin, Dominique Desplancq, Audrey Stoessel, Christian Massute, Jeremy Ranniger, Alastair G. McEwen, Gabrielle Zeder-Lutz, Mustapha Oulad-Abdelghani, Manuela Chiper, Pierre Lafaye, Barbara Di Ventura, Pascal Didier, Arnaud Poterszman and Etienne Weiss

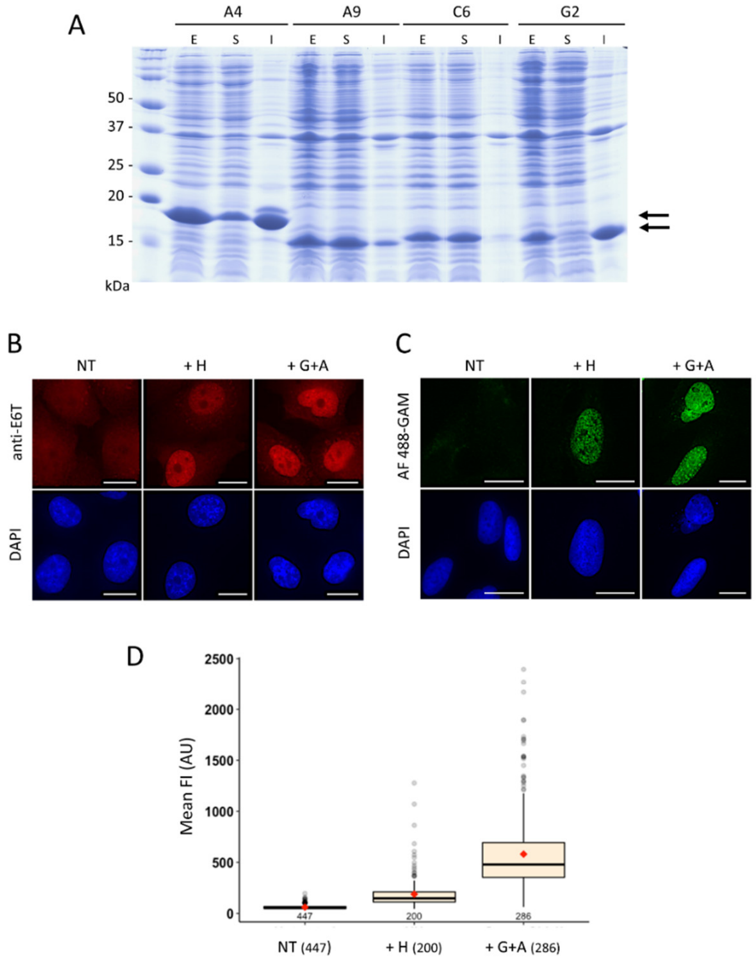

Figure S1. Characterization of the A9 nanobody. (**A**) Analysis by SDS-PAGE of the bacterially-expressed nanobodies. The gel shows the protein content of similar amounts of total (E), soluble (S) and insoluble (I) fractions of extracts obtained after lysis of the induced bacteria. The bands corresponding to the nanobody polypeptides are indicated with arrows. (**B** and **C**) Representative immunofluorescence images of drug-treated H1299 cells recorded after incubation with either A9 nanobody (B) or mAb 3F4 (C). Scale bar: 20 μm. (**D**) Quantification of the signal obtained with the cells shown in (C). The number of analyzed cells is indicated in brackets.

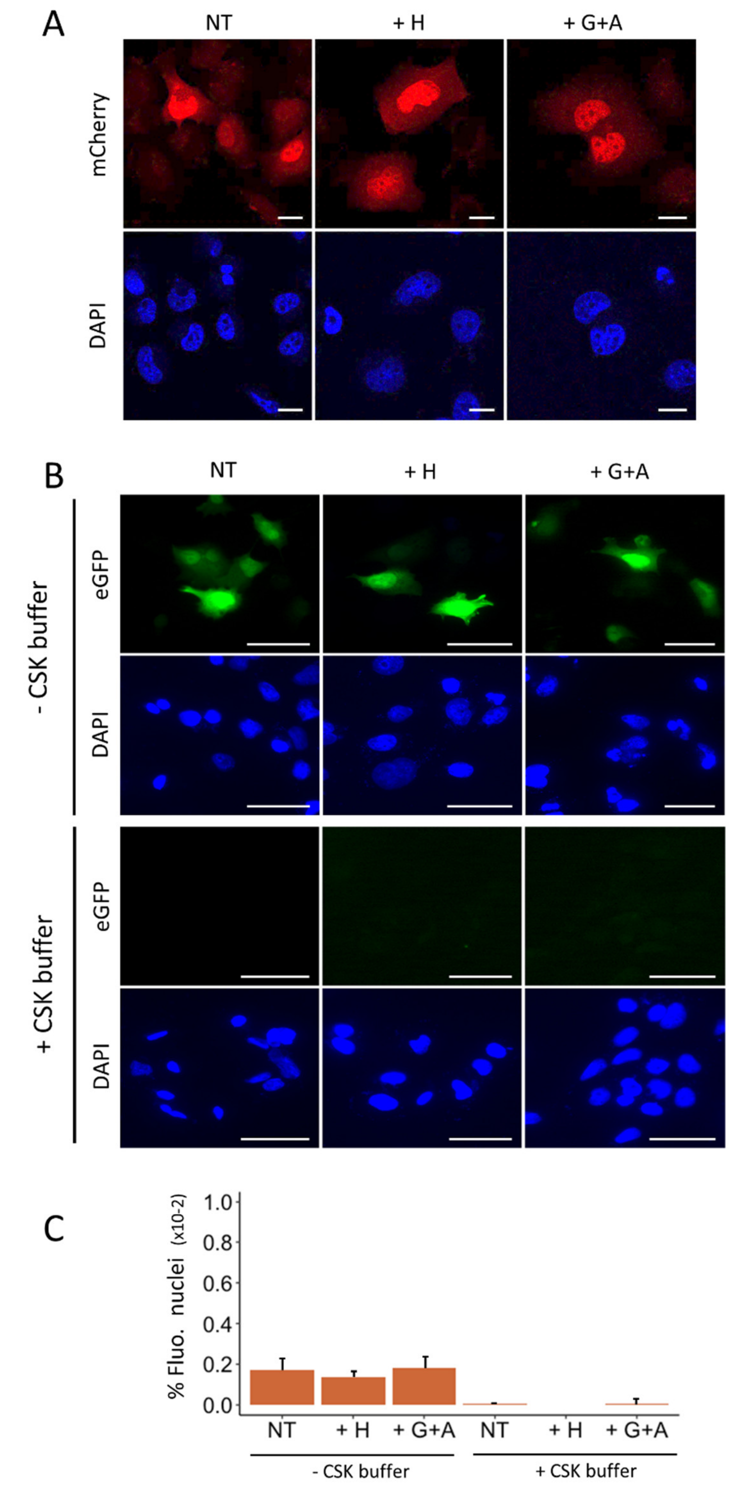

Figure S2. Microscopic analysis of C6 and A9 nanobodies. (**A**) Representative confocal microscopy images of H1299 cells after transfection of the C6 nanobody-mCherry construct. The cells were treated as indicated in the legend of Fig. 2C. Scale bar: 20 µm. (**B** and **C**) Immunofluorescence analysis of H1299 cells after transfection with chromobody A9-GFP. The cells were treated as indicated in the legend of Fig. 3B. Representative images recorded under the microscope (**B**) and the corresponding percentage of fluorescent cells observed in each condition (**C**) are shown. Cut-off for negative cells was set on non-transfected cells using the maximal recorded value. Scale bar: 50 µm.

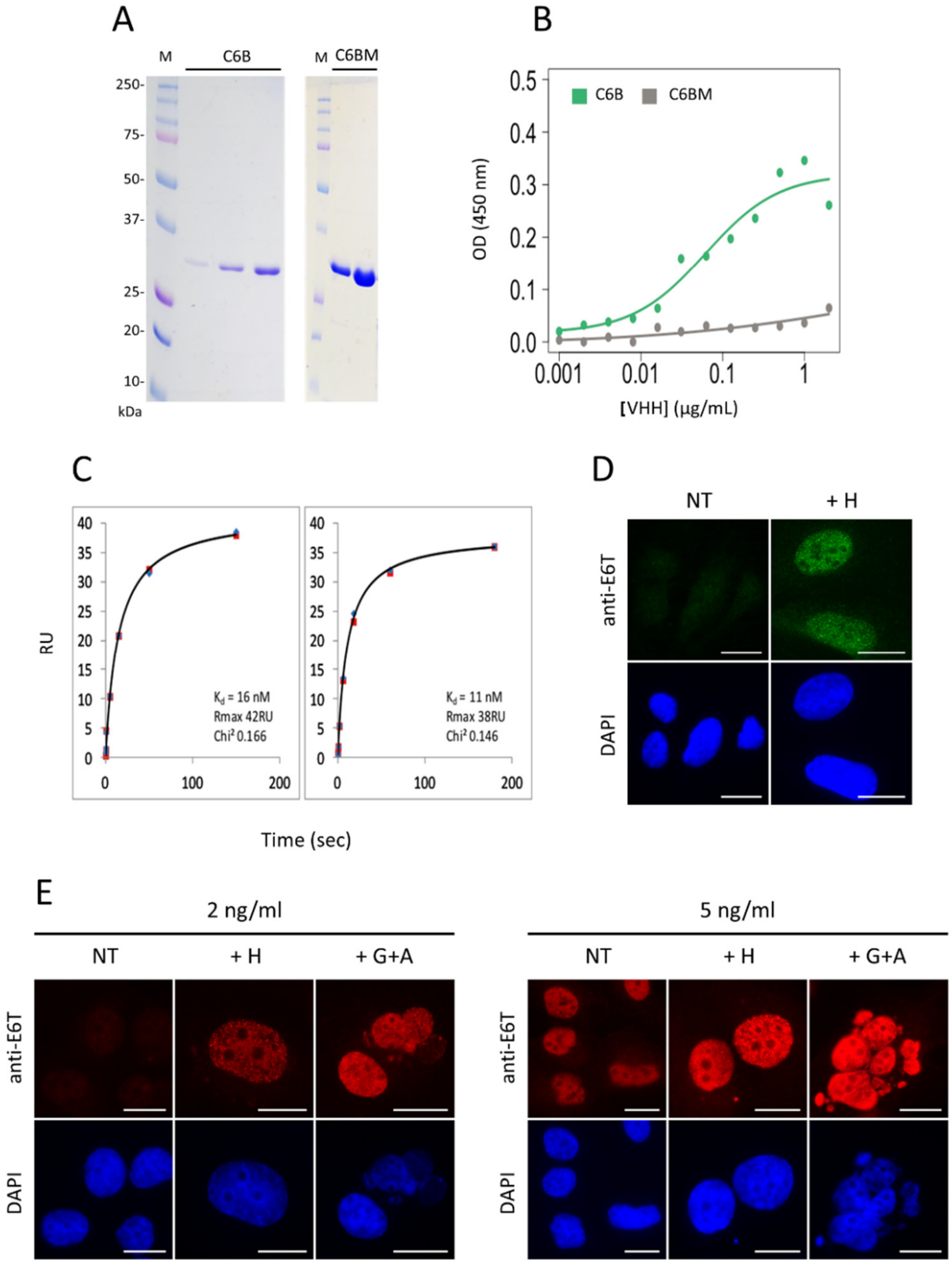

Figure S3. Biochemical and fluorescence microscopic analyses of C6B an C6BM nanobodies. (**A**) Purification and analysis on SDS gel of the C6B and C6BM nanobodies. Aliquots of affinity-purified protein samples (1 to 5 µg) were subjected to SDS-PAGE and subsequent Coomassie blue staining. (**B**) Varying concentrations of C6B and C6BM nanobodies were probed by ELISA with fixed phospho-peptide on plate (0.1 µg/mL). (**C**) Typical binding profiles of the C6 nanobody to the phospho-peptide as probed by SPR (Materials and Methods). The experimental values of each experiment are indicated. (**D**) Immunofluorescence assay with the C6B nanobody in U2OS cells. Bound nanobodies were revealed with anti-E6 tag antibodies and Alexa Fluor 488 anti-mouse immunoglobulins. The nuclei were counterstained with DAPI (blue). Scale bar: 20 µm. (**E**) Representative immunofluorescence images of H- or G+A-treated H1299 cells following fixation and incubation with 2 ng/mL or 5 ng/mL of bivalent C6B nanobody bound material was revealed with anti-E6T antibody and Alexa 568-labelled anti-mouse globulins. The nuclei were counterstained with DAPI (blue). Scale bar: 20 µm.

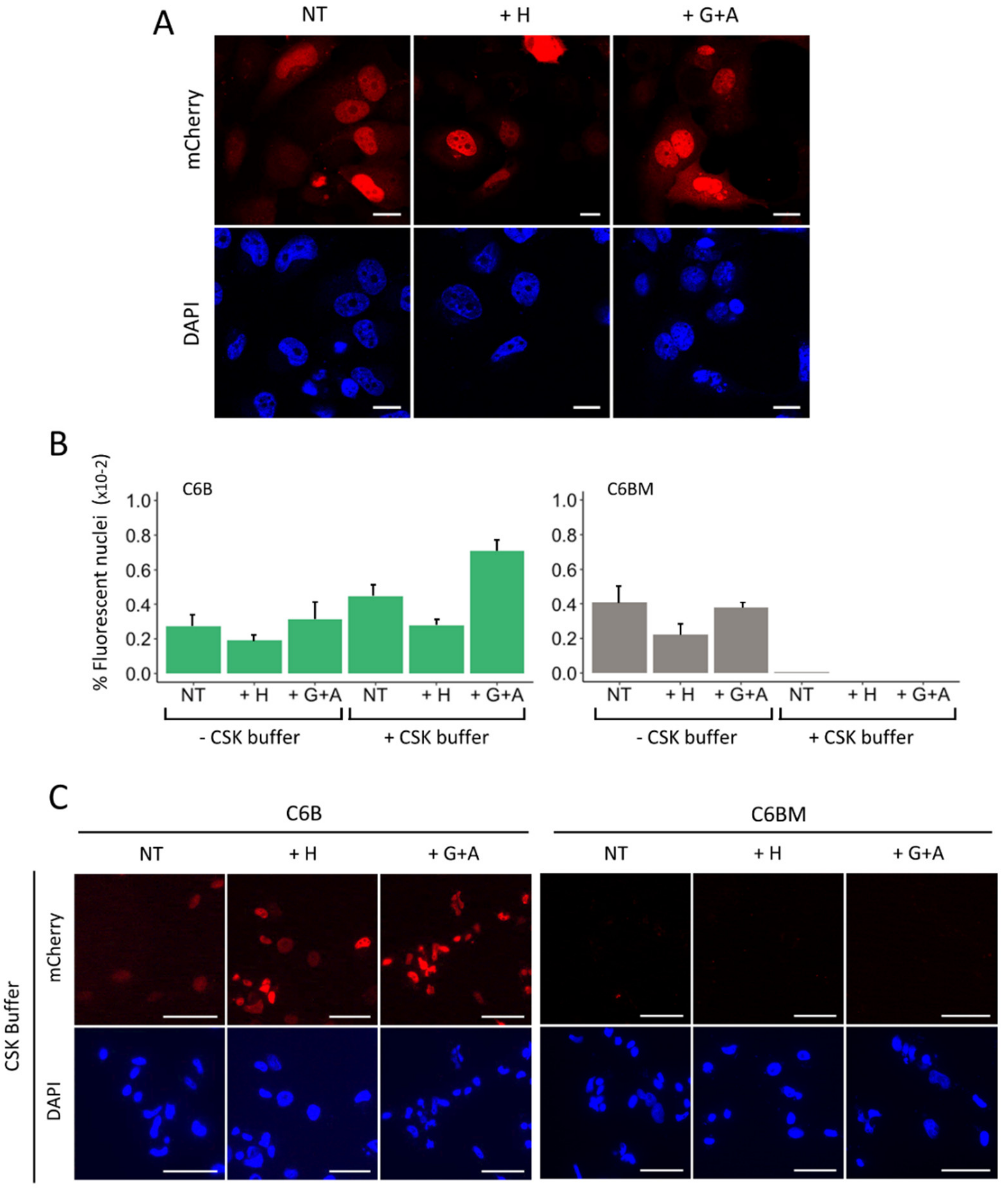

Figure S4. Expression of the bivalent chromobodies in transfected cells. (**A**) Representative immunofluorescence images of H1299 cells transfected with the pβA-C6B-E6T-mCherry construct. The transfected cells were treated with the indicated drugs for 24 h and after cell fixation, expressed nanobody-mCherry fusions were monitored under a confocal microscope. Scale bar: 20 µm. (**B**) Evaluation of the binding stability of the C6B-mCherry or the C6BM-mCherry fusions expressed in H1299 cells treated with the indicated drugs after transfection. The histograms show the percentage of fluorescent nuclei detected after fixation (- CSK buffer) or after a wash with CSK buffer prior to fixation (+ CSK buffer). Up to 300 nuclei recorded from 3 independent experiments in each condition were analyzed to calculate the percentages. (**C**) Representative immunofluorescence images of the transfected H1299 cells used in (**B**). Scale bar: 50 µm.

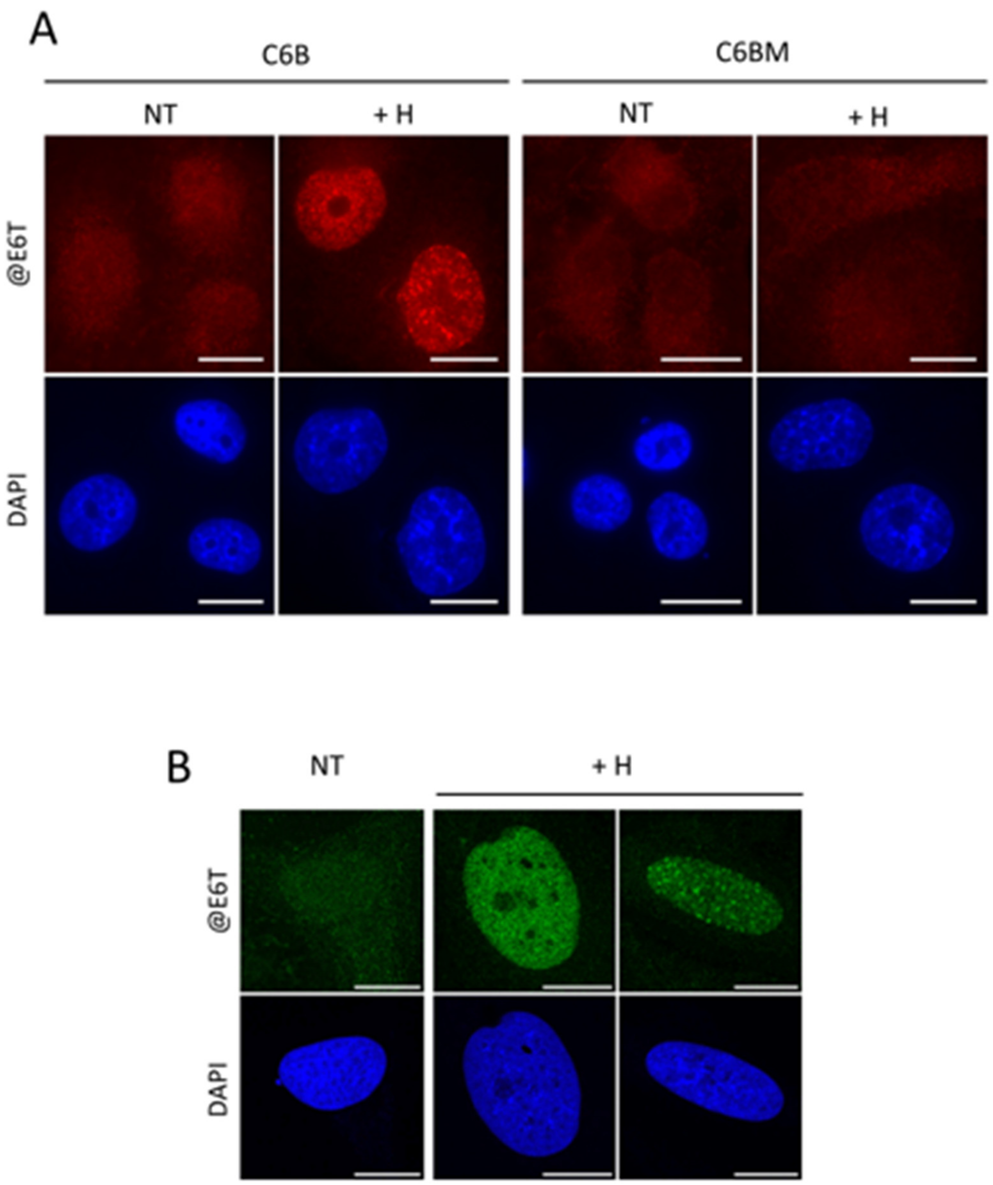

Figure S5. Transduction of the C6B or C6BM nanobodies in cancer cells. (**A**) Transduction of C6B and C6BM in H1299 cells. Representative images recorded by fluorescence microscopy after treatment of the cells with H or left untreated (NT). The nanobodies were revealed as indicated in the legend of Figure 4. Scale bar: 20 µm. (**B**) Transduction of C6B nanobodies in U2OS cells treated as in (**A**). Representative images taken with a confocal microscope after DAPI counterstaining (blue) are shown. The C6B molecules were revealed as indicated in (**D**) of Figure S3. Scale bar: 10 µm.

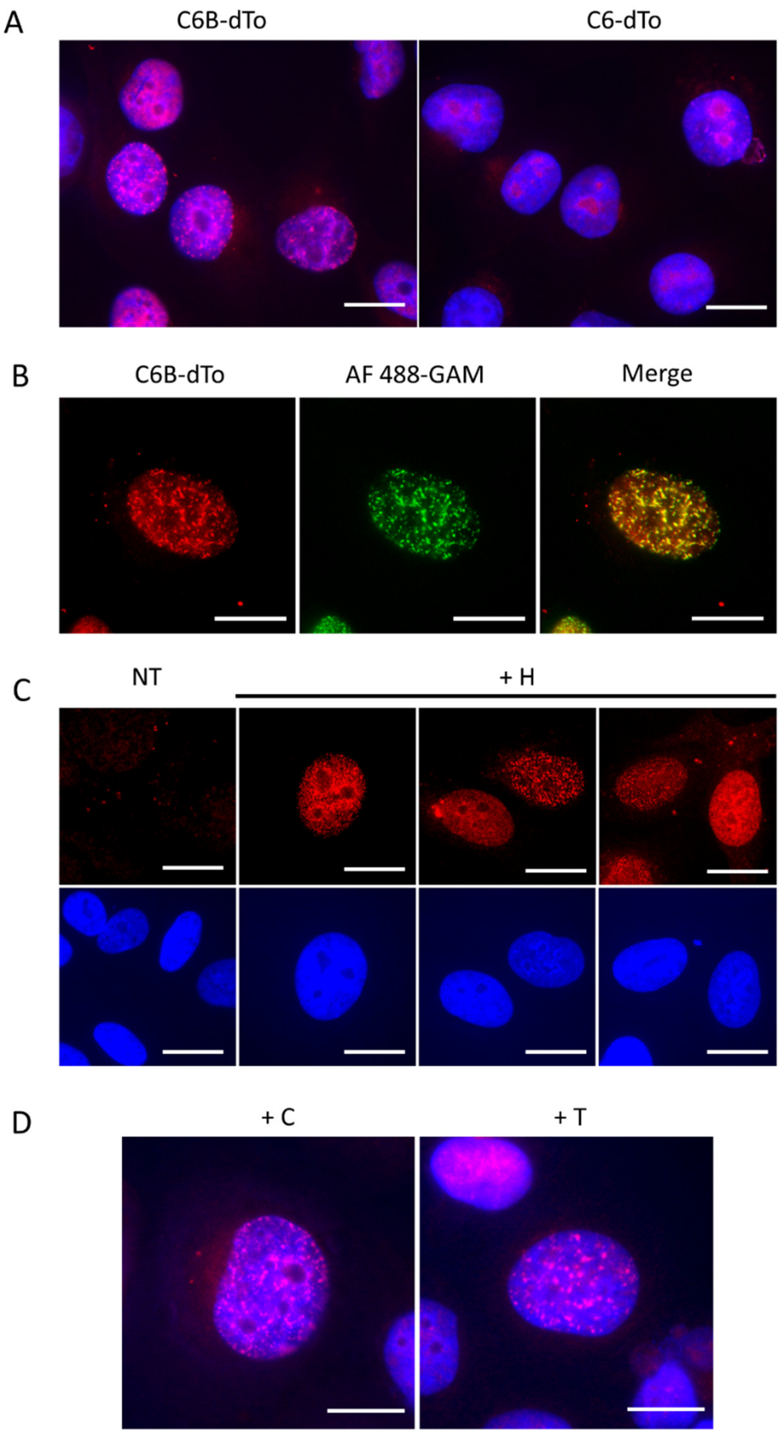

Figure S6. Specific detection of foci with the C6B-dTo chromobody. (**A**) Comparison of the binding performance of C6-dTo and the C6B-dTo chromobodies. Equivalent amounts of monovalent or divalent chromobodies were delivered in H1299 cells and images were taken after treatment of the transduced cells for 24 h with H. The pictures shown correspond to composite images of the dTomato and the DAPI signals. Scale bar: 20 µm. (**B**) H1299 cells were transduced with the C6B-dTo chromobody and treated as indicated in (**A**). Prior to analysis by immunofluorescence microscopy, they were incubated with mAb 3F4 and Alexa 468-labelled secondary anti-mouse globulins. The pictures show the foci pattern of a typical cell following analysis with red (left) or the green (middle) filters. Yellow color shows the colocalization of the chromobody and the mAb at foci (right). Scale bar: 10 µm. (**C**) Transduction of C6B-dTo chromobodies in U2OS cells treated as in (**A**). Scale bar: 20 µm. (**D**) Detection of foci in H1299 cells transduced with C6B-dTo chromobody and subsequent treatment with either clofarabine (C) or triapine (T). Typical nuclei after analysis as in (**A**) are shown. Scale bar: 10 µm.

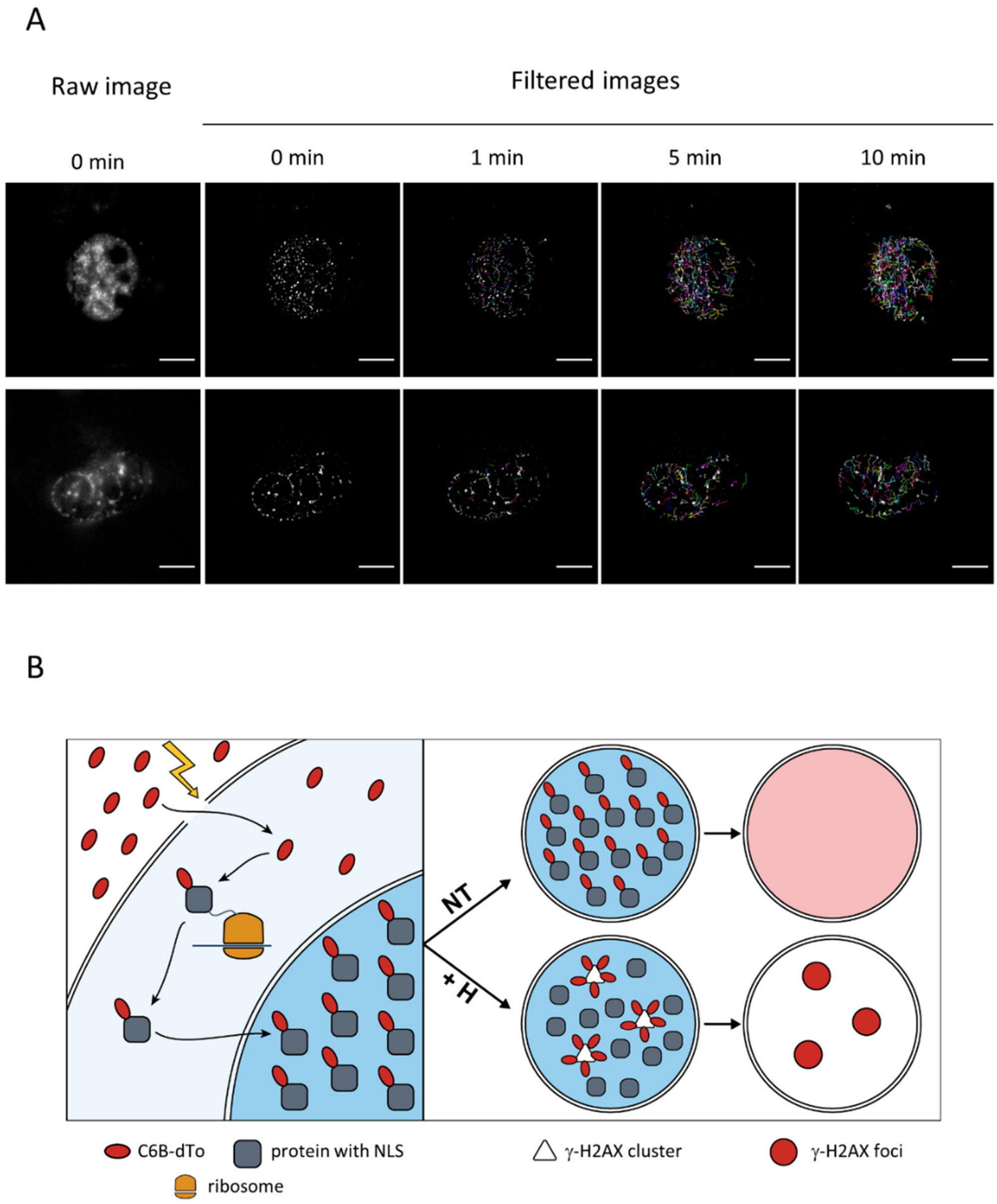

Figure S7. Visualization of the movement of the C6B-dTomato molecules in live H1299 cells and schematic representation of their binding in drug-injured cells. (**A**) Two representative wide-field fluorescence microscopy images of H1299 cells transduced with the C6B-dTomato fusion protein and subsequently treated with G+A during 4 h (left) were analyzed as indicated in the legend of Figure 6. The trajectories of the γ-H2AX foci over a period of 10 minutes are shown. Scale bar: 10 µm. (**B**) The left panel represents the internalization and nuclear transport of the C6B-dTo molecules. Upon delivery in the cytoplasm by electroporation they bind to newly-synthesized nuclear proteins (grey square) and are piggybacked in the nucleus (blue compartment). The accumulation of the C6B-dTo molecules at discrete sites upon treatment with H and γ-H2AX foci formation in the nucleus is shown in the right panel. Without drug treatment, the C6B-dTo molecules remain homogeneously distributed in the nucleus and the faint speckled staining observed after transduction is almost no more visible upon cell division.

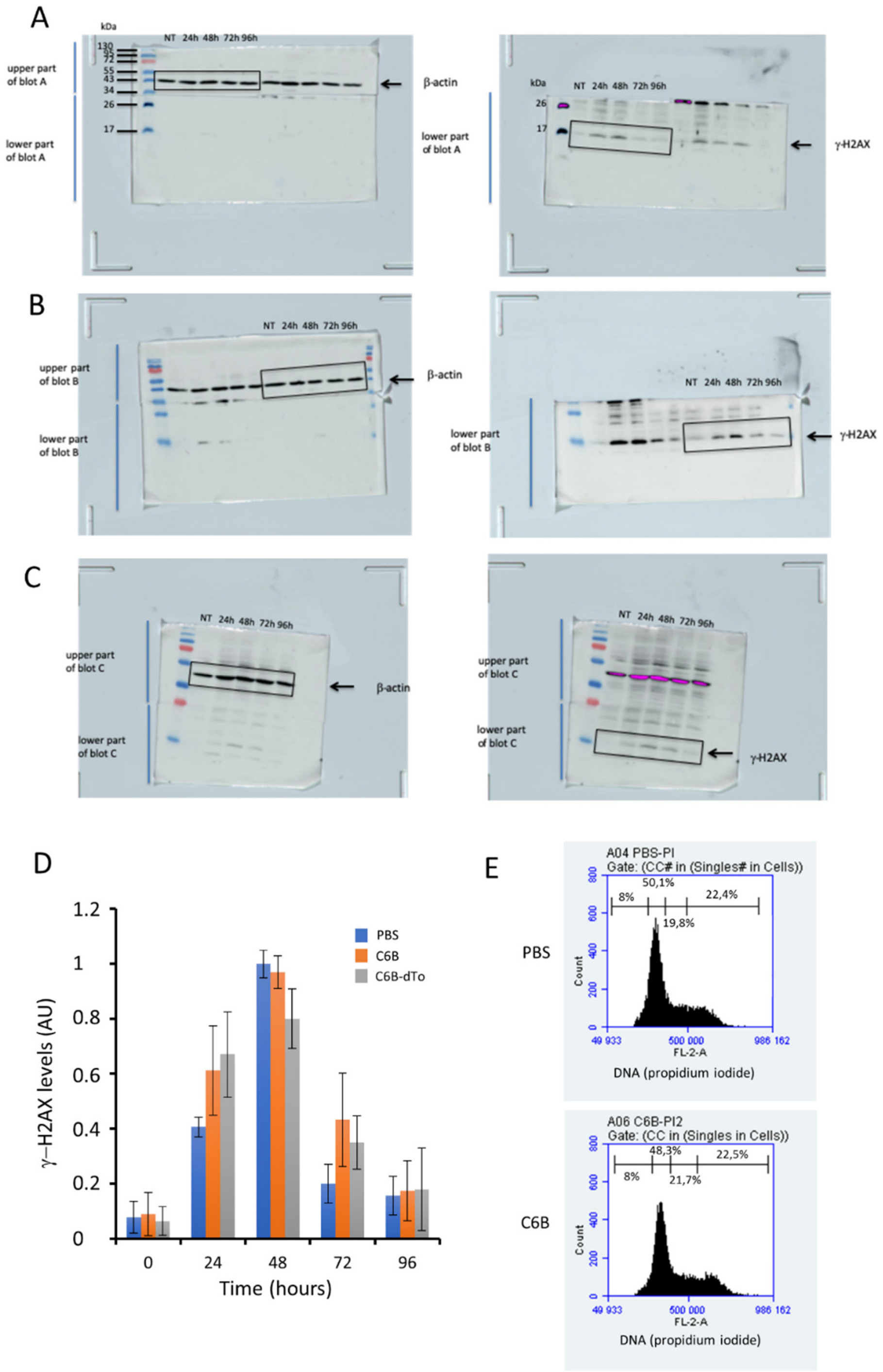

Figure S8. Western blot and FACS analyses of the transduced H1299 cells after pulse-treatment with hydroxyurea. (**A**–**C**) The pictures show the original blots used for constructing Figure 6D. After transduction with either PBS (**A**), nanobody C6B (**B**) or chromobody C6B-dTo (**C**), the cells were pulse-treated with hydroxyurea for 24 hours and whole-cell extracts prepared at the indicated timepoints were subjected to Western blot analysis with either mAb 3F4 (lower part of the membrane) or with anti-β-actin antibodies (upper part of the membrane). The boxed areas correspond to the signals used for Figure 6D. (**D**) Quantification of the γ-H2AX levels shown in A-C. The data correspond to mean values of the luminescent intensity of the γ-H2AX bands recorded from 3 independent experiments after normalization. (**E**) FACS analysis of the cells transduced with either PBS or C6B 24 hours after removal of hydroxyurea (48 h timepoint on the blots). Approximately 10^6 cells were counted in each case. The panels correspond to the measurement of the DNA content. The percentage of cells in sub-G1, G1, S and G2 is indicated.

Table S1. Data collection and refinement statistics of the C6 nanobody in complex with the peptide corresponding to the γ-H2AX tail.

**Data Collection**	
Resolution range (Å)	61.49-1.55 (1.605-1.55)
Space group	P 3_1
a, b, c (Å)	87.41, 87.41, 105.41,
α, β, γ (°)	90, 90, 120
Total No. reflections	1376552 (137008)
No. of unique reflections	130630 (13033)
Multiplicity	10.5 (10.5)
Completeness (%)	99.77 (99.90)
‹*I*/*σ*(*I*)›	13.47 (0.99)
R-merge	0.0903 (1.938)
R-meas	0.0949 (2.038)
R-pim	0.0291 (0.627)
*CC*1/2	0.999 (0.679)
*CC**	1 (0.899)
Overall B factor from Wilson plot (Å²)	21.48
**Structure Refinement**	
No. of reflections used in refinement	130626 (13030)
No. of reflections used for test set	6402 (651)
R-work	0.1491 (0.3091)
R-free	0.1844 (0.3268)
CC(work)	0.976 (0.877)
CC(free)	0.955 (0.860)
Number of non-hydrogen atoms	7621
Macromolecules	6621
Ligands	8
Solvent	992
R.M.S. deviations	
Bonds (Å)	0.006
Angles (°)	0.86
Ramachandran plot	
Most favored (%)	98.95
Allowed (%)	1.05
Outliers (%)	0
Average B-factor (Å²)	31.81
Macromolecules	30.29
Ligands	36.24
Solvent	41.9
Rotamer outliers (%)	1.75
Clashscore	0.99

Table S2. List of the primers used for the construction of the VHH libraries and for generating the pET- and pβ-actin-based expression vectors.

Name	Nucleotide Sequence	Used for Generating
VHBACK-A6	5′-GATGTGCAGCTGCAGGCGTCTGGRGGAGG-3′	VHH-PCR1
CH2FORTA4	5′-CGCCATCAAGGTACCAGTTGA-3′	VHH-PCR1
CALL001 (ref)	5′-GTCCTGGCTGCTCTCTACAAGG-3′	VHH-PCR1
CALL002 (ref)	5′-GGTACGTGCTGTTGAACTGTTCC-3′	VHH-PCR1
AlpVh-L (ref)	5′-CTGAGCTTGGTGGTCCTGGCTGC-3′	VHH-PCR1
Bq-lead-lg-F	5′-GTCCTGGCTGCTCTWYTACARGG-3′	VHH-PCR1
Bq-CH2-ca2-R	5′-GGTACGTGCTGTTGAACTGTTCC-3′	VHH-PCR1
SM017	5′-CCAGCCGGCCATGGCTCAGGTGCAGCTGGTGGAGTCTGG-3′	VHH-PCR1
SM018	5′-CCAGCCGGCCATGGCTGATGTGCAGCTGGTGGAGTCTGG-3′	VHH-PCR1
VHBACKA4	5′-CATGCCATGACTCGGGGCCCAGCCGGCCATGGCGAKGTSCAGCT-3′	VHH-PCR2
VHFOR36	5′-GGACTAGTTGCGGCCGCTGAGGAGACGGTGACCTG-3′	VHH-PCR2
Bq-FR1-long-F	5′-GTCATTGGCCCAGCCGGCCATGGCTCAGKTGCAGCTCGTGGAGTCNGG-3′	VHH-PCR2
Bq-Hin-short-F	5′-GACATTGCGGCCGCGCTGGGGTCTTCGCTGTGGTG-3′	VHH-PCR2
Bq-Hin-long-R	5′-GACATTGCGGCCGCTGGTTGTGGTTTTGGTGTCTTGGG-3′	VHH-PCR2
E6T-For	5′-CTAGTATGTTTCAGGATCCAGAACGTCCGCGCG-3′	pETOM
E6T-Back	5′-CTAGCGCGCGGACGTTCCTGCGGATCCTGAAACATA-3′	pETOM
VHH-BspH1-For	5′-AACGAACTCATGACTCAGKTGCAGCTCGTGGAGTCGGG-3′	pET-C6B
VHH-BspH1-Deg	5′-AACGAACTCATGACYSABBTSCAGCTSSWGSMGTCVCC-3′	pET-C6B
VHH-Not1-short	5′-GGACTAGTTGCGGCCGCTGAGGAGACGGTGACCTG-3′	pET-C6B
VHH-Not1-long	5′-GGACTAGTTGCGGCCGCTGGTTGTGGTTTTGGTGTTTCGGG-3′	pET-C6B
pETOM-For	5′-GGAGACCACAACGGTTTCCC-3′	pETOM
pET-Rev	5′-TTCGGGCTTTGTTAGCAGCC-3′	pETOM
b-actin-For	5′-GGCTCACAGCGCGCCCGGCT-3′	pET-C6B
G4S-Rev	5′-CGATCCGCCACCGCCGCTGCCACCTCCGCCTGAACCGCCTCCACCGGCC GCTGAGGAGACGGTGA-3′	pET-C6B
G4S-For	5′-GGTGGAGGCGGTTCAGGCGGAGGTGGCAGCGGCGGTGGCGGATCGATG ACTGAGGTGCAGCTGGT-3′	pET-C6B
E6Tag-Rev	5′-TCCTGCGGATCCTGAAACAT-3′	pET-C6B
C6-Cys-Rev	5′-AAAAAATGCGGCCGCACATGAGGAGACGG-3′	pET-C6B
C6-Mut-For	5′-GCAGATATACCGCGATACAGTCTGAG-3′	pET-C6M
C6-Mut-Rev	5′-CTCAGACTGTATCGCGGTATATCTGC-3′	pET-C6M
mCher-Rev	5′-CTTGTACAGCTCGTCCATGCC-3′	pET-C6
mCher-pET-For	5′-ATTTATGCTAGCGGAGGGATGGTGAGCAAGGGC-3′	pET-C6B-mCherry
mCher-pET-Rev	5′-ATTTATGCTAGCACTACCCTTGTACAGCTCGTCC-3′	pET-C6B-mCherry
dTo-Bam-For	5′-GCGCATGGATCCTATGGTGAGCAAGGGCGAGGAG-3′	pET-C6B-dTo
dTo-Bam-Rev	5′-GCGCGCGGATCCCCGGTGCTGCCGGTGCCATG-3′	pET-C6B-dTo
E6T-Hind-For	5′-CTAGTATGTTTCAGGATCCGCAGGAACGTCCGCGCAAGCTTG-3′	pbA-VHH-ET
E6T-Hind-Rev	5′-CTAGCAAGCTTGCGCGGACGTTCCTGCGGATCCTGAAACATA-3′	pbA-VHH-ET
mCherry-For	5′-ATTTATAAGCTTAGTGGGATGGTGAGCAAGGGC-3′	pbA-VHH-ET-mC
mCherry-Rev	5′-ATTTATGAATTCTCATTACTTGTACAGCTCGTCC-3′	pbA-VHH-ET-mC
NESPKIa-Hind-For	5′-AGCTTAACGAGCTCGCTCTCAAACTCGCTGGACTCGACATCAACAAGACCA-3′	pbA-C6B
NESPKIa-Hind-Rev	5′-AGCTTGGTCTTGTTGATGTCGAGTCCAGCGAGTTTGAGAGCGAGCTCGTTA-3′	pbA-C6B

Legends to videos

Video S1. Analysis by time-lapse microscopy of a nucleus of H1299 cells transduced with C6B-dTomato proteins (0.5 µg) and treated with H. Images were taken as indicated in the legend of Figure 6. Scale bar: 10 µm.

Video S2. Analysis by time-lapse microscopy of a nucleus of H1299 cells transduced with C6B-dTomato proteins (2 µg) and treated with H for 24 h. Images were taken as indicated in the legend of Figure 6. Scale bar: 10 µm.

Video S3. Analysis by time-lapse microscopy of a nucleus of H1299 cells transduced with C6B-dTomato proteins (2 µg) and treated with G+A for 24 h. Images were taken as indicated in the legend of Figure 6. Scale bar: 10 µm.

Video S4. Analysis by time-lapse microscopy of a nucleus of H1299 cells transduced with C6B-dTomato proteins (2 µg) and treated with H for 24 h. Images were taken and processed as indicated in the legend of Figure 6. Scale bar: 10 µm.

Video S5. Analysis by time-lapse microscopy of a nucleus of H1299 cells transduced with C6B-dTomato proteins (2 µg) and treated with H for 24 hours. Images were taken and processed as indicated in the legend of Video S4. Scale bar: 10 µm.

Video S6. Analysis by time-lapse microscopy of a typical nucleus of H1299 cells transduced with C6B-dTomato proteins (2 µg) and treated with G+A for 4 h. Images were taken and processed as indicated in the legend of Figure 6. The Scale bar: 10 µm.

Video S7. Analysis by time-lapse microscopy of a nucleus of H1299 cells transduced with C6B-dTomato proteins (2 µg) and treated with G+A for 4 hours. The g-H2AX pattern observed in this case may correspond to a nucleus in mid-S phase. Images were taken and processed as indicated in the legend of Figure 6. Scale bar: 10 µm.
